# Profiling the Genomes and Secreted Effector Proteins in *Phytopythium vexans* Global Strains

**DOI:** 10.3390/jof11070477

**Published:** 2025-06-23

**Authors:** Oscar Villanueva, Hai D. T. Nguyen, Walid Ellouze

**Affiliations:** 1 London Research and Development Centre, Agriculture and Agri-Food Canada, 4902 Victoria Avenue North, Vineland, ON L0R 2E0, Canada; 2 Department of Biological Sciences, Brock University, 1812 Sir Isaac Brock Way, St. Catharines, ON L2S 3A1, Canada; 3 Ottawa Research and Development Centre, Agriculture and Agri-Food Canada, 960 Carling Avenue, Ottawa, ON K1A 0C6, Canada

**Keywords:** oomycete, functional genomics, pathogenomics, effector proteins, virulence factors

## Abstract

*Phytopythium vexans* is a plant pathogen responsible for a variety of destructive diseases in crops worldwide. This includes patch canker, damping-off, root, and crown rots in economically important crops, such as apple, pear, grapevine, citrus, avocado, and kiwi. The pathogen has a global distribution, and a recent report confirmed its presence in southern Ontario, Canada. This study presents the first genome sequencing, assembly, and annotation of the Canadian *P. vexans* strain SS21. To explore how variation in secreted protein repertoires may relate to infection strategies and host adaptation, we compared the predicted secretome of SS21 with reference strains from Iran (CBS 119.80) and China (HF1). The analysis revealed that HF1 harbors a larger set of CAZymes, sterol-binding proteins, and predicted effectors, which may suggest broader adaptive potential. In contrast, strain SS21 appears to have adapted to a niche-specific strategy, with fewer necrosis-inducing proteins, glucanase inhibitors, and effectors, possibly indicating adaptation to specific hosts or ecological conditions. Comparative genome data highlight distinct evolutionary trajectories that may have shaped each strain’s infection strategy, with SS21 potentially serving as a robust additional reference for future studies on *P. vexans* biology and host interactions. While this analysis identifies key candidate effectors, gene expression studies are required to validate their functional roles in infection and host manipulation.

## 1. Introduction

The *Phytopythium* genus comprises approximately 28 species, several of which have been recently identified as destructive plant pathogens [1]. Among these, *Phytopythium vexans* has been reported as responsible for root and crown rot, as well as the decline of economically important fruit trees, woody ornamentals, and cultivated crops [1]. This includes apple and pear in Morocco [2], apple and rubber trees in China [3,4], avocados in the Canary Islands [5], citrus, apple, and peach in Tunisia [6,7], durian in Australia and Vietnam [8,9], ginkgo, red maple, Fraser fir and flowering cherry in USA [10,11,12], grapevines, eucalypts and pines in South Africa [13,14], kiwifruit in Turkey and Italy [15,16], strawberry in the Czech Republic [17], raspberry in Scotland [18] and common bean in Rwanda [19]. Recently, this pathogen was identified for the first time in Ontario, Canada [20], an indication of its wide distribution and threat to global agriculture.

The life cycle of *P. vexans* involves several stages, each closely associated with the secretion of effector proteins that facilitate infection. The cycle begins with zoospores, which exhibit chemotaxis, moving toward optimal infection sites on host plants [21]. On the plant cell surface, encysted zoospores germinate, forming germ tubes and appressoria, structures that penetrate the plant cuticle. Once inside the host, *P. vexans* produces haustoria, specialized intracellular infection structures. Haustoria are enveloped by a plant-derived membrane known as the extrahaustorial membrane, which serves as the critical interface between plant and pathogen. The extra haustorial membrane facilitates nutrient uptake and acts as a platform for the pathogen to deliver virulence factors that manipulate the host’s cellular defenses [22]. By deploying effector proteins through the haustoria, *P. vexans* effectively disarms the plant defenses, promoting infection and disease progression.

**Figure 1:**
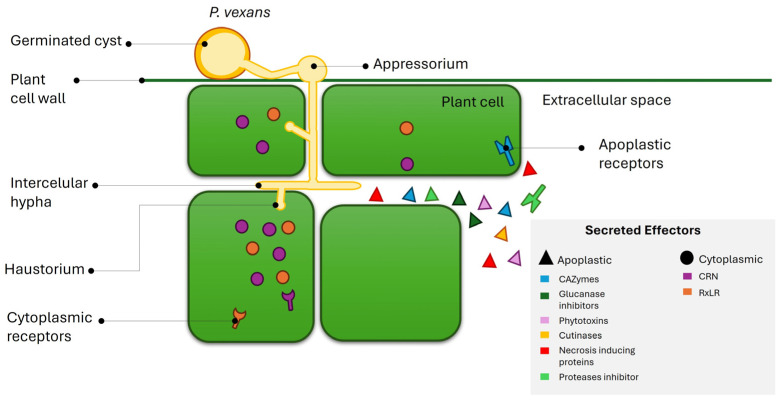
Schematic representation of effector secretion from *P. vexans* haustorium and intracellular hyphae. Apoplastic effectors (triangles) are secreted into the extracellular space, where they interfere with apoplastic plant defenses. Cytoplasmic effectors (circles) translocate inside host cells, thereby crossing two membranes, one from the pathogen and another from the host.

Effector proteins are broadly defined as molecules secreted by pathogens that promote virulence, elicit resistance, or cause cytotoxicity to facilitate disease [23]. To be classified as a secreted effector protein, a molecule typically possesses a signal peptide for secretion, lacks transmembrane domains, is small in size, and is often species-specific [24,25]. Effectors can be secreted into the host cytoplasm (cytoplasmic effectors) or the extracellular space (apoplastic effectors) (Figure 1).

Effector proteins are crucial in infection, manipulating the host at the infection interface to the pathogen’s advantage. Identifying and characterizing pathogen effector repertoire is an important step in understanding the molecular mechanisms that drive disease incidence and severity. The increasing availability of sequenced oomycete genomes has enabled detailed studies of how these pathogens cause devastating crop losses. Computational prediction of secreted proteins from genomic sequences is a valuable tool for narrowing down candidate effectors for experimental validation [25,26,27].

*Phytopythium vexans* secretes both cytoplasmic and apoplastic effectors. The two major classes of cytoplasmic effectors in oomycetes are Crinkler (CRN) and RxLR. Both classes have a secretion signal at the N-terminus and one or more domains toward the C-terminus. CRN proteins contain an LxLFLAK motif and a highly conserved “HVLV” motif, distinguishing the N-terminus from the C-terminus. While these motifs are characteristic of many CRN effectors, their presence is not strictly required for functionality, as some effectors lacking one or both can still retain activity. The CRN protein family is widespread among oomycetes and is thought to be more ancient than RxLR effectors [26,28,29]. RxLR effectors are the most studied group of cytoplasmic effectors characterized by the RxLR motif (arginine, any amino acid, leucine, arginine) and a second conserved motif termed EER (glutamate, glutamate, arginine) toward the C-terminus; however, studies have shown that some effectors lacking one or both motifs can still enter host cells and remain functional [30]. Apoplastic effectors, like their cytoplasmic counterparts, contain a signal peptide within the first 60 amino acids at the N-terminus, followed by one or more domains toward the C-terminus [31]. These effectors are typically small and rich in cysteine residues, similar to serine or cysteine protease inhibitors. Oomycete apoplastic effectors include numerous hydrolytic enzymes, such as cutinases, glycoside hydrolases, pectinases, proteases, and CAZymes, which degrade host cell components to facilitate infection [32]. Additionally, oomycetes may encode extracellular toxins such as necrosis-inducing proteins (NLPs) and Pcf family toxins [33].

To date, limited *P. vexans* genome sequences are available in the National Center for Biotechnology Information (NCBI), and they include *P. vexans* HF1 [34], CBS 119.80 = DAOM BR484 [28,35], and the Canadian strain SS21 (GenBank: GCA_023337965.1), which is reported here for the first time. While the secretome of various oomycetes has been studied [24,34,36,37], a thorough evaluation of a Canadian strain of *P. vexans* has not been conducted.

This study provides the first genome sequence, assembly, and annotation of the Canadian *P. vexans* SS21 strain and a comparative secretome analysis with reference strains from Iran (CBS 119.80) and China (HF1). We hypothesize that *P. vexans* strains isolated from different geographic regions and hosts differ in their infection strategies and host adaptation, as reflected in variations in secretome composition, particularly in cytoplasmic and apoplastic effectors. We also provide a comprehensive overview of the *P. vexans* secretome, offering insights into genes potentially involved in pathogenicity.

## 2. Materials and Methods

### 2.1. DNA Extraction and Genome Sequencing

*Phytopythium vexans* SS21 (deposited at the Canadian Collection of Fungal Cultures as DAOMC 252529) was isolated from southern Ontario soil in a commercial orchard with a known history of tree fruit root rot disease [20]. *P. vexans* SS21 mycelia were grown in 5% V8^®^ original vegetable cocktail juice (Campbell, ON, Canada) broth for 10 days at 25 °C. The V8^®^ broth was prepared by adding 1 g of CaCO_3_ to 100 mL of V8^®^ original vegetable cocktail juice, followed by centrifugation at 6000× *g* for 10 min. Fifty ml of the V8 supernatant was mixed with 950 mL of sterile distilled water to create the V8 broth. The broth was sterilized at 120 °C for 20 min before use. Mycelia grown in liquid medium were separated by filtration through sterilized Whatman^™^ Qualitative filter paper grade 1 (Cytiva, Marlborough, MA, USA), washed with sterile distilled water, and freeze-dried for 48 h using a benchtop freeze drying system (Labconco FreeZone^®^ 4.5 Liter, Kansas City, MO, USA). Genomic DNA was extracted from SS21 mycelia following the manufacturer’s protocol using the DNeasy^®^ PowerSoil^®^ Pro Kit (Qiagen, MD, USA, cat. #47016). DNA quality was evaluated with a DeNovix spectrophotometer (DeNovix Inc., Wilmington, NC, USA). The genomic DNA library was prepared and sequenced using the Illumina NovaSeq 6000 PE150 platform at (Genome Quebec, Montreal, QC, Canada).

### 2.2. Genome Assembly

The genome of *P. vexans* SS21 was assembled using Illumina short reads (available at ENA: www.ebi.ac.uk/ena/browser/view/SRR18156462, accessed on 21 October 2024). Illumina reads were trimmed using the ‘bbduk.sh’ script from the BBMap package v38.22 (available at SourceForge: https://sourceforge.net/projects/bbmap/, accessed on 21 October 2024). The trimming process involved removing adapters and low-quality bases with the following parameters: ‘ref = adapters qtrim = rl trimq = 20 minlength = 36 ktrim = r forcetrimleft = 15 tossjunk = t’. The trimmed reads were then assembled into a genome using MEGAHIT v1.2.9 [38] with default parameters (k-mer sizes: 21, 29, 39, 59, 79, 99, 119, 141). The resulting contigs were ordered by length, and those shorter than 1000 bp were discarded. The final assembly of *P. vexans* SS21 was uploaded to NCBI genomes, where it was automatically screened for contamination. To assess the completeness of the genome assembly, BUSCO analyses were performed with BUSCO v5.4.3 [39], running in genome mode. Genome assembly statistics were calculated using QUAST v5.0.2 [40].

### 2.3. Phylogenetic Analyses

The genome of strain SS21 was sequenced, assembled, and annotated in this study, while the genomes of 16 additional *Phytopythium* spp. and *Halophytophthora polymorphica* CBS 680.84 were downloaded from NCBI. Forty-eight core genes, detailed in Table S1, were extracted, aligned, and concatenated using the Universal Fungal Core Genes (UFCG) pipeline version 1.0.5 [41]. Maximum Likelihood-based phylogenetic analysis of the concatenated sequences was performed using IQ-Tree 2.2.6 [42], with 1000 bootstrap replicates conducted to determine the best-scoring Maximum Likelihood (ML) tree. The phylogenetic tree was rooted with *H. polymorphica* CBS 680.84.2.4.

### 2.4. Genome Annotation

Genome annotation was performed on the decontaminated assembly of SS21 with funannotate v1.8.13 (https://github.com/nextgenusfs/funannotate, accessed on 15 January 2024) following the standard procedure outlined in the official documentation (https://funannotate.readthedocs.io/en/latest/, accessed on 15 January 2024). In brief, assemblies were first repeat-masked with Tantan v39 [43]. Coding sequences (CDS) were extracted from the *P. vexans* HF1 genome (NCBI Genomes WGS record QLOC00000000.1, https://www.ncbi.nlm.nih.gov/Traces/wgs/QLOC01, accessed on 21 October 2024), which had been re-annotated in a previous study [35], and used as supporting transcript evidence. The ab initio gene prediction was performed following a methodology similar to that described in the same study [35]. Transcript evidence from HF1 was mapped to the masked genome using Minimap2 v2.24 [44]. Subsequently, Diamond v2.0.15 [45] and Exonerate v2.4.0 [46] were employed to map a set of pre-downloaded proteins from the UniProtKB/Swiss-Prot database (released in March 2022, containing 568,002 sequences) [47] to the masked genome assembly. BUSCO v2.0 [48] was then used to identify conserved gene models for training ab initio predictors. The gene prediction process continued with Augustus v3.3.2 [49], SNAP (version from 28 July 2006) [50], and GlimmerHMM v3.0.4 [51] to generate gene models. EVidenceModeler (EVM) v1.1.1 [52] was used to combine the gene models from the ab initio predictions into a final set. The ‘funannotate predict’ command was executed with the following additional options: ‘--min_training_models 50’, ‘--busco_db alveolata_stramenophiles’, and ‘--organism other’.

Genome annotations (protein files) of CBS 119.80 (NCBI Genomes WGS record JAADXV000000000.1, https://www.ncbi.nlm.nih.gov/Traces/wgs/JAADXV01, accessed on 21 October 2024) and HF1 (NCBI Genomes WGS record QLOC00000000.1, https://www.ncbi.nlm.nih.gov/Traces/wgs/QLOC01, accessed on 21 October 2024) performed in Nguyen et al. [35] were downloaded for subsequent comparative analyses with SS21 of this study. To assess the completeness of the genome annotation of CBS 119.80, HF1, and SS21 strains, BUSCO analyses were performed using the stramenopiles_odb10 database with BUSCO v5.4.3 [39], running in protein mode.

Gene orthology analysis was conducted using OrthoVenn3 (bioinfotoolkits.net, accessed on 21 October 2024), where orthologous analysis was performed on predicted *P. vexans* protein files with the OrthoMLC algorithm, setting the E-value threshold to 1e-15.

### 2.5. Identification of Putative Effectors

All predicted proteins for each of the *P. vexans* strains were analyzed using InterProScan 5. Proteins identified with Pfam domains associated with pathogenicity were classified as potential effectors based on criteria established in previous studies [31].

To further characterize apoplastic effectors, the predicted proteins of the *P. vexans* strains CBS 119, HF1, and SS21 were submitted to the dbCAN3 web server for automated carbohydrate-active enzyme (CAZyme) annotation (https://bcb.unl.edu/dbCAN2/, accessed on 21 October 2024). The protein sequences were analyzed using HMMER: dbCAN (E-value 1e-15, coverage > 0.35), DIAMOND: CAZy (E-value < 1e-102), and HMMER: dbCAN-sub (E-value < 1e-15, coverage > 0.35). Additionally, the remaining apoplastic proteins were predicted using the InterProScan database with default settings, incorporating Phobius, SignalP, SMART, and TMHMM into the analysis. The results were filtered based on transmembrane domain prediction and the presence of signal peptides using TMHMM and SignalP, respectively. Proteins with an HMM S probability value of ≥0.9, an NN Ymax score of ≥0.5, and an NN D score of ≥0.5, predicted to be secreted with no transmembrane domain following the signal peptide cleavage site, were considered putatively secreted.

The prediction of RxLR and CRN cytoplasmic effectors was conducted using the effectR tool (effectR/README.md at master · grunwaldlab/effectR · GitHub). This tool identifies candidate effectors through a two-step process. First, it conducts regular expression (REGEX) searches to detect canonical motifs: RxLR and EER for RxLR effectors and LxLFLAK and HVLV for CRN effectors. Second, it applies Hidden Markov Model (HMM) profile searches to detect proteins that show structural similarity to known RxLR or CRN effectors, even if they lack recognizable motifs. Candidate effectors were classified into four categories based on motif presence: (i) complete, containing both motifs; (ii) only RxLR or LxLFLAK; (iii) only EER or HVLV; and (iv) no motifs. The “no motifs” group refers to sequences that matched the HMM profile but did not contain canonical motifs and may represent divergent effectors or partial gene predictions. To ensure accuracy, the predicted proteins were then validated by running them through the InterProScan database [53].

## 3. Results

### 3.1. Genome Assembly, Gene Modeling and Phylogeny

The genome statistics are summarized in Table 1. Genome sizes were 35.3 Mbp for SS21, 41.73 Mbp for HF1, and 33.85 Mbp for CBS 119.80, with 2843, 44, and 3685 contigs assembled, respectively. HF1 exhibited the largest contig assembled (3.17 Mbp) and the highest N50 value (1.51 Mbp), indicating superior assembly contiguity compared to SS21 (N50: 31.5 kbp) and CBS 119.80 (N50: 29.2 kbp).

**Figure 2:**
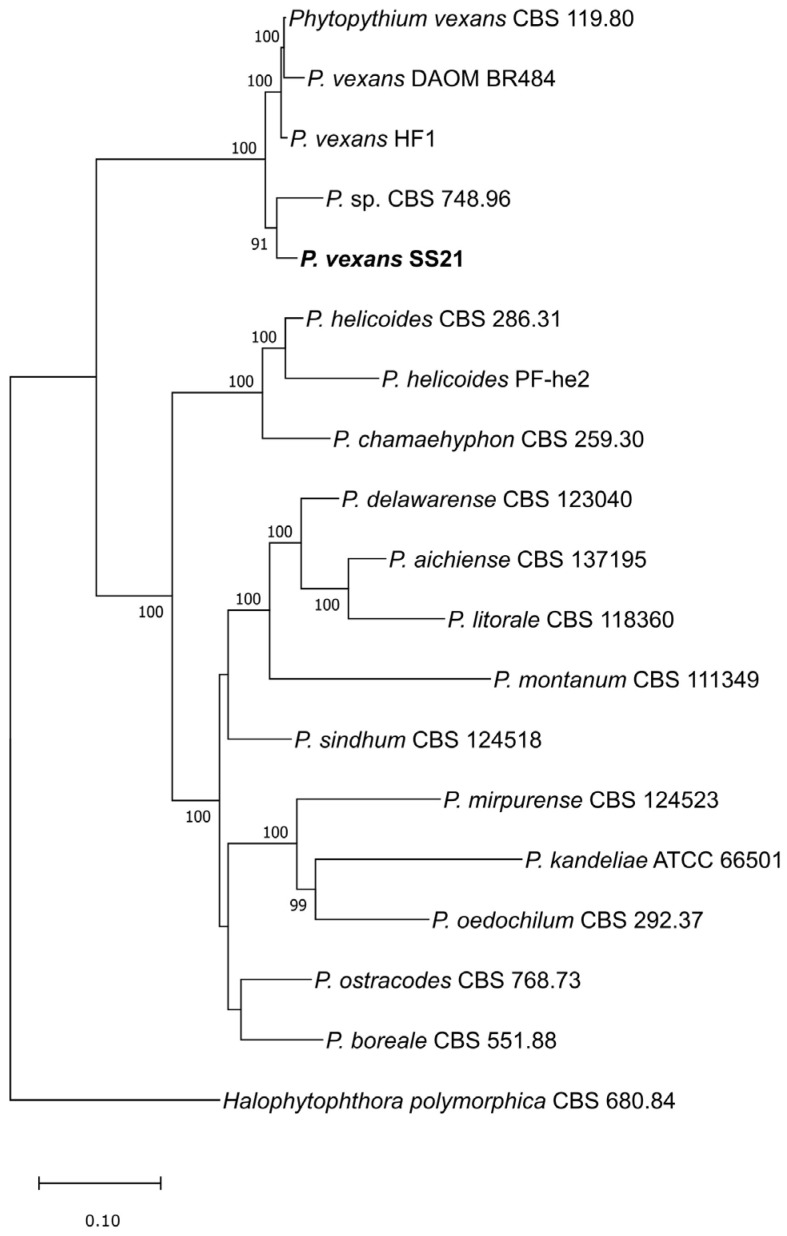
Maximum likelihood phylogenetic tree generated using a concatenated alignment of 48 core genes from 17 *Phytopythium* spp. strains sourced from NCBI. The *P. vexans* SS21 genome from this study is highlighted in bold. Bootstrap values greater than 50 are shown at each node. The tree was rooted to *H. polymorphica* CBS 680.84. The numbers at each node represent bootstrap support, expressed as percentages. Only bootstrap values greater than 50% are shown.

Genome completeness, as determined by BUSCO analysis, was highest in SS21 (99.0%), followed by HF1 (92.2%) and CBS 119.80 (90.6%). The genome coverage was 412× for SS21, 87× for HF1, and 78× for CBS 119.80. Predicted gene counts were consistent across the strains, with HF1 containing 14,537 genes, SS21 with 12,619, and CBS 119.80 with 12,693. BUSCO protein completeness was uniformly high, at 99% for all strains. The maximum likelihood (ML) phylogenetic tree (Figure 2) was constructed using a concatenated alignment of 48 core genes extracted from the whole genomes of the 17 *Phytopythium* spp. available in NCBI, along with the genome of *Halophytophthora polymorphica* CBS 680.84, which was used as an outgroup to root the tree. The phylogenetic analysis confirmed the identification of the Canadian strain SS21, which clustered monophyletically with other *P. vexans* strains, including CBS 119.80, DAOM BR484, two independent assemblies of the same Iranian strain, and HF1. *Phytopythium* sp. CBS 748.96 strain clustered monophyletically with *P. vexans* strains, including SS21. However, it was excluded from the secretome analysis because it was deposited under the invalid name *Phytopythium cucurbitacearum* (nom. inval.), and its taxonomic status remains unresolved.

### 3.2. Secretome Analysis

In this study, we compared the predicted secretome of the recently reported Canadian *P. vexans* strain SS21 with strains CBS 119.80 and HF1. Proteins were predicted to contain a signal peptide and lack a transmembrane domain after the signal peptide was considered secreted. Out of the 12,619, 12,693, and 14,537 predicted proteins for SS21, CBS 119.80, and HF1, respectively, 7.30% (920 proteins) in SS21, 8.12% (1031 proteins) in CBS119.80, and 8.14% (1223 proteins) in HF1 were predicted to be secreted. These putative secreted proteins were classified based on their InterPro and Pfam domains, specifically searching for those associated with pathogenicity. The proteins were grouped into three categories: microbe-associated molecular patterns (MAMPs), apoplastic effectors, and cytoplasmic effectors (Table 2).

### 3.3. Microbe-Associated Molecular Pattern (MAMP) Genes

Among the *P. vexans* strains analyzed, HF1 contains the highest number of genes encoding sterol-binding proteins (57), followed by CBS 119.80 (52) and SS21 (42). These proteins are essential for sterol acquisition and crucial for the survival and pathogenicity of sterol-auxotrophic oomycetes. Additionally, all strains reported the same number of transglutaminase proteins (4).

### 3.4. Apoplastic Effectors Profiling

**Figure 3:**
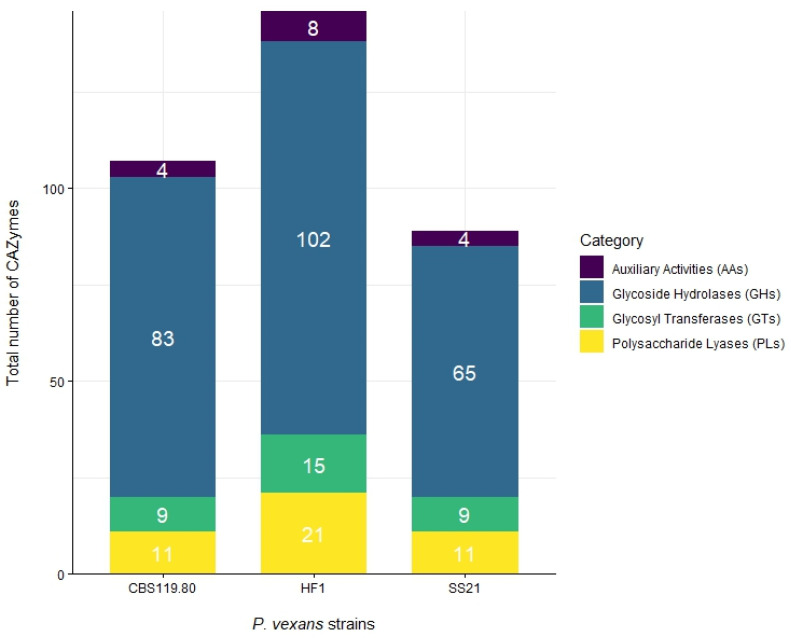
Total number and distribution of CAZymes across *P. vexans* strains CBS 119.80, HF1, and SS21.

From the predicted secretome, proteins were annotated to identify Carbohydrate-Active Enzymes (CAZymes). Figure 3 illustrates the distribution of genes encoding CAZymes in the three *P. vexans* strains examined in this study: CBS 119.80, HF1, and SS21.

**Figure 4:**
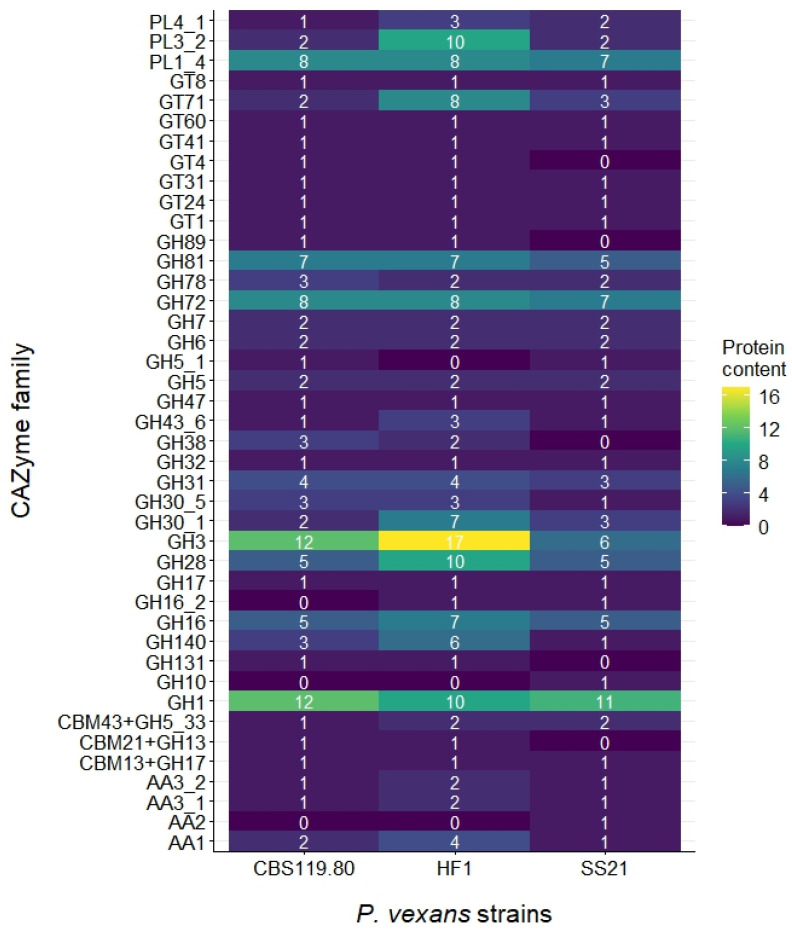
Comparative abundance of CAZyme families across *P. vexans* SS21, CBS 119.80, and HF1. Glycoside hydrolases (GHs), glycosyl transferases (GTs), polysaccharide lyases (PLs), carbohydrate esterases (CEs), auxiliary activities (AAs), and carbohydrate-binding modules (CBMs).

Glycoside hydrolases (GHs) are pivotal for carbohydrate metabolism, facilitating the hydrolysis of glycosidic bonds in complex carbohydrates. HF1 showed an expanded set of GH3 (17) and GH1 (10) family members associated with cellulose degradation (Figure 4), compared to CBS 119.80 (12 GH1, 12 GH3) and SS21 (11 GH1, 6 GH3), which may suggest enhanced enzymatic potential. Additionally, GH28 enzymes involved in pectin degradation were more abundant in HF1 (10) than in CBS 119.80 and SS21 (5 each). GH140 enzymes, responsible for breaking down complex polysaccharides, were also more abundant in HF1 (6) than in CBS 119.80 (3) and SS21 (1).

Glycosyl transferases (GTs) are crucial for the transfer of glycosyl groups, playing a key role in the biosynthesis of polysaccharides and glycoproteins. The HF1 strain contains 15 GTs, surpassing the 9 GTs found in both CBS 119.80 and SS21 (Figure 3). In addition, the GT71 family, which could be involved in the synthesis of glycoproteins and polysaccharides that contribute to the structure and integrity of the cell wall, is more abundant in HF1 (8 GTs) compared to CBS 119.80 (2 GTs) and SS21 (3 GTs) (Figure 4).

Polysaccharide lyases (PLs) degrade polysaccharides via β-elimination mechanisms. HF1 contained 21 PLs, whereas CBS 119.80 and SS21 each had 11 PLs (Figure 3), suggesting a greater enzymatic capability for polysaccharide breakdown in HF1.

Auxiliary activities (AAs), involved in the oxidative degradation of polysaccharides, were least represented. HF1 had 8 AAs, while CBS 119.80 and SS21 each had 4 (Figure 3), indicating a preference for hydrolytic over oxidative mechanisms in these strains.

Other CAZyme families exhibited varied representation. The GH10 family, involved in xylan degradation, was only present in SS21 with 1 count (Figure 4). The GH16 and GH16_2 families, slightly more abundant in HF1 (7 GH16 and 1 GH16_2), are associated with β-1,3-glucanase activity. The CBM21 + GH13 family was present in CBS119.80 and HF1, consistently low across those strains, while the AA1, involved in lignin and phenolic compound degradation, were slightly more abundant in HF1 (4), followed by CBS119.80 (2) and SS21 (1) (Figure 4).

Overall, HF1 encoded the largest number of CAZymes among the strains analyzed, including an expanded set of GHs associated with cellulose and pectin degradation and GTs linked to glycosylation processes.

Glucanase inhibitors protect the pathogen from plant glucanases. We found six inhibitors in SS21 and CBS 119.80 each, while HF1 had 12 (Table 2), indicating a potentially enhanced capacity in HF1 to counteract plant defense mechanisms.

Necrosis-inducing proteins trigger programmed cell death in host plants. HF1 exhibited higher counts of 52 versus 28 in SS21 and CBS 119.80.

### 3.5. Cytoplasmic Effectors Profiling

No phytotoxins or cutinases were detected in any of the three strains, indicating these proteins may not play significant roles in the pathogenic strategies of *P. vexans*.

**Figure 5:**
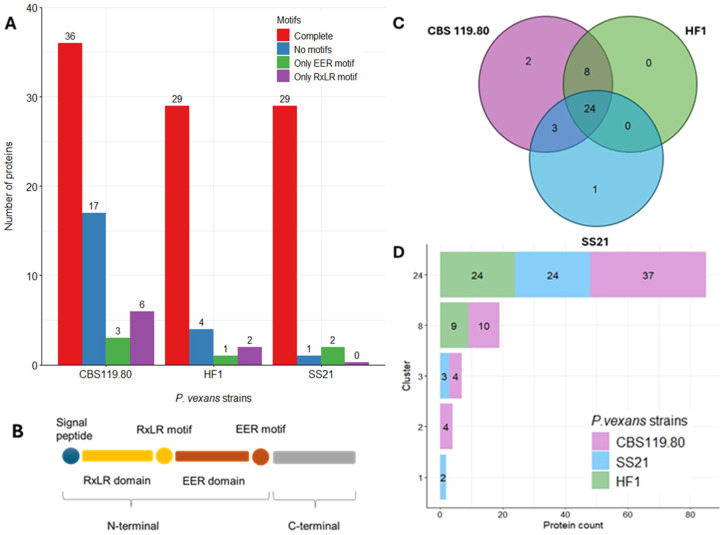
RxLR motif content across *P. vexans* strains. (**A**) Categories of RxLR motifs: “complete” (RxLR + EER motifs), “only RxLR”, “only EER”, and “no motifs” (HMM hit lacking both motifs). (**B**) Schematic of RxLR effectors showing signal peptide, RxLR, and EER motifs. (**C**) Comparison of RxLR orthologous protein clusters. (**D**) Protein count per ortholog cluster.

RxLR effectors are a critical class of proteins in the pathogenicity of oomycetes, renowned for their capability to manipulate host cells and facilitate infection [23]. RxLR effectors were identified where CBS 119.80 had the highest number of RxLR effectors with complete motifs (36), followed by HF1 (29) and SS21 (29) (Figure 5A,B).

CBS 119.80 also exhibited a substantial number of effectors lacking motifs (17), whereas HF1 and SS21 had fewer such effectors (4 and 1, respectively). Effectors with only RxLR or EER motifs were minimal across all strains (Figure 5A,B).

Figure 5C,D shows that 24 RxLR clusters, based on protein similarity, are shared among all three strains, indicating a conserved core set of RxLR effectors. Additional clusters were shared between CBS 119.80 and HF1 (eight clusters) and between CBS 119.80 and SS21 (three clusters). Unique clusters were identified in CBS 119.80 (two clusters) and SS21 (one cluster), suggesting a degree of strain-specific diversification.

**Figure 6:**
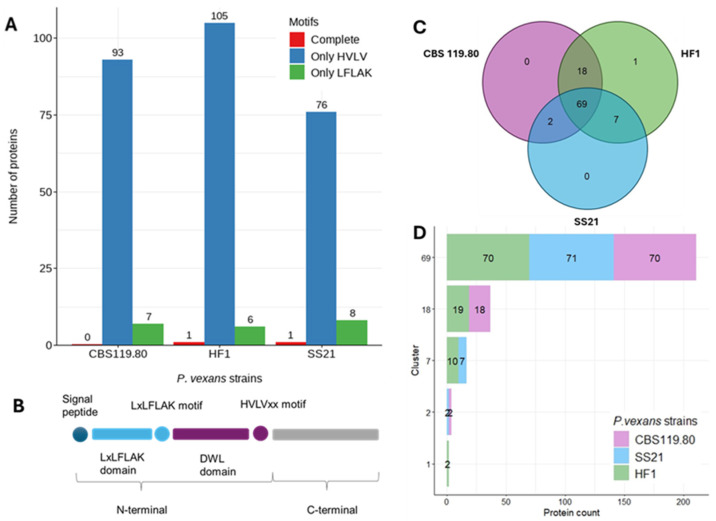
CRN motif content in *P. vexans* strains. (**A**) Distribution of CRN effectors containing both LxLFLAK and HVLVxx motifs, only LxLFLAK, or only HVLVxx. (**B**) CRN effector structure showing signal peptide, LxLFLAK, and HVLVxx motifs. (**C**) Comparison of CRN ortholog clusters among strains. (**D**) Protein count per cluster.

CRN effectors (Figure 6A,B), another major class of cytoplasmic effectors, were analyzed for motif presence. Across all strains, the ‘Only HVLV’ motif was most prevalent: CBS 119.80 (93 sequences), HF1 (105), and SS21 (76). Sequences containing only the LFLAK motif were rare: CBS 119.80 (7), HF1 (6), and SS21 (8). Complete motifs (both LFLAK and HVLV) were exceptionally rare, with only one sequence in HF1 and SS21.

Figure 6C,D illustrates that 69 CRN clusters, based on protein similarity, are common to all strains, with additional shared clusters between CBS 119.80 and HF1 (18) and between SS21 and HF1 (7). Unique clusters were present in CBS 119.80, SS21 (2 each), and HF1 (1), suggesting both conserved and strain-specific CRN effector functions.

## 4. Discussion

Phylogenomic analysis confirms the identification of SS21 as *P. vexans* since it clusters with other strains of this species (Figure 2). HF1 had superior assembly contiguity, likely due to the added PacBio reads, emphasizing the importance of long sequencing technology in achieving high-quality and more contiguous genome assemblies. However, SS21’s higher BUSCO genome completeness, consistent gene count, and high BUSCO protein completeness suggest that SS21 could serve as an additional robust reference for future comparative studies.

Elicitins, or sterol-binding proteins, are highly conserved and crucial for sterol-auxotrophic oomycetes like *P. vexans*, which acquire sterols from host plants due to their inability to synthesize them [54]. Elicitins are also vital for maintaining cell membrane integrity and influencing pathogenicity [55]. Among *P. vexans* strains, HF1 has the highest number of sterol-binding protein genes at 57, followed by CBS 119.80 with 52 and SS21 with 42. Elicitins were less abundant in SS21, which may indicate a reduced capacity for sterol acquisition in this strain. This limitation could potentially affect its survival and pathogenicity under certain environmental conditions. Similar sterol-binding protein counts have been noted in other oomycetes like *Phytophthora cactorum* [26]. However, the presence of a larger number of predicted sterol-binding proteins does not necessarily correlate with a broader host range or increased pathogenicity. Elicitins such as INF1 can function as avirulence factors by triggering host immune responses, suggesting that eliciting expansion may, in some cases, restrict rather than enhance host compatibility. In contrast, the number of transglutaminase proteins is consistent across *P. vexans* strains, suggesting a conserved role in fundamental biological processes rather than involvement in strain-specific adaptations.

The comparative analysis of CAZymes across *P. vexans* strains HF1, CBS 119.80, and SS21 shows that HF1 has the highest number of CAZymes at 146, followed by CBS 119.80 at 107 and SS21 at 89. Glycoside hydrolases, essential for carbohydrate metabolism for catalyzing the hydrolysis of glycosidic bonds in complex carbohydrates [56], are most abundant in HF1 (102 GHs). Glycosyl transferases, involved in polysaccharide and glycoprotein biosynthesis [28,57], are most frequent in HF1 (15 GTs). HF1 shows a higher abundance of polysaccharide lyases (21 PLs), which cleave polysaccharides through β-elimination [58], and auxiliary activities (8 AAs), involved in oxidative polysaccharide degradation. This suggests that HF1 has a broader array of enzymes for complex carbohydrate degradation compared to CBS 119.80 and SS21, which may rely more on hydrolytic enzymes for polysaccharide breakdown [58,59]. However, further validation is needed to confirm these predictions.

Specific families such as GH3 (β-glucosidase activity, McGowan and Fitzpatrick [31]), GH28 (pectin degradation, Villarreal et al. [60]), GT71 (glycosylation, Breton et al. [57]), GH16 (β-1,3-glucanase activity crucial for cell wall degradation [61]), and pectate lyases like PL1_4 and PL3_2 [62] are particularly abundant in HF1. On the other hand, the CBM21 + GH13 family, involved in starch and glycogen degradation, shows a low presence across all strains, suggesting a minor role in their carbohydrate metabolism [63]. The AA1 and AA2 families, involved in lignin and phenolic compound degradation, are similarly distributed among the strains, indicating comparable lignin degradation capabilities [59]. While the abundance of glycosyl hydrolases in HF1 may suggest an enhanced capacity for plant cell wall degradation, it is important to note that such enzymes are also common in saprophytic and non-pathogenic fungi, where they function primarily in nutrient acquisition. Therefore, the presence of these enzymes alone does not necessarily imply pathogenicity. Gene expression levels should be assessed to determine their functional relevance during infection.

Overall, HF1 had the highest count of predicted CAZymes, which may indicate a greater potential for complex carbohydrate degradation and glycosylation compared to the other strains. However, it is important to note that a higher number of predicted enzymes does not necessarily translate into increased enzymatic activity or functional capacity without further experimental validation. Despite having fewer CAZymes overall, both SS21 and CBS 119.80 maintain robust sets of GHs and GTs, indicating that they also play important roles in carbohydrate metabolism and potentially in virulence. HF1’s broader enzyme repertoire is consistent with findings in other oomycetes, such as *Phytophthora* species, where expansions in CAZyme families, particularly GHs, are linked to enhanced pathogenicity and host adaptation [64,65]. HF1’s higher number of GHs for cellulose and pectin breakdown corroborates expansions observed in oomycetes like *Phytophthora* and *Aphanomyces*, where elevated GH counts facilitate efficient carbohydrate degradation. However, species-specific adaptations emphasize unique functional roles. For example, *Aphanomyces astaci* has expanded GH18 chitinases to break down chitin in crayfish exoskeletons [31], a function absent in *P. vexans*, which focuses on plant-derived carbohydrate degradation. This highlights the diversity of GHs across oomycetes, shaped by their respective hosts and ecological niches.

SS21 and CBS 119.80 produce six glucanase inhibitors, fewer than the 12 in HF1, suggesting a reduced ability to counteract plant defenses. SS21 also has fewer necrosis-inducing proteins (27 NLPs) compared to HF1 (52 NLPs), indicating a potentially less aggressive infection strategy. Studies in other oomycetes have shown that *Phytophthora* species harbor large numbers of NLPs, with expansions linked to their pathogenic adaptability. *Phytophthora sojae* has 80 NLPs, while *Ph. ramorum* and *Ph. parasitica* contain 69 and 74 NLPs, respectively [31]. In contrast, *Pythium* species tend to have fewer NLPs, with most species possessing fewer than seven copies [31], reflecting specialized infection strategies. *Phytopythium vexans* SS21’s limited NLPs, and glucanase inhibitors point to a more targeted, niche-specific approach to infection rather than broad-spectrum pathogenicity.

The lower number of RxLR and CRN effectors observed in *P. vexans* SS21 compared to CBS 119.80, and HF1 aligns with evolutionary trends seen across various oomycetes. In *Ph. capsici*, RxLR, and CRN effectors are often clustered in rapidly evolving, gene-sparse regions of the genome, rich in transposons and repetitive elements [66]. These regions are hotspots for evolutionary changes, which enable pathogens to adapt quickly to host immune responses [27,67]. The reduced number of effectors in SS21, particularly those with complete motifs, may reflect a more streamlined set of effectors specialized for certain hosts or ecological conditions, a phenomenon observed in other oomycetes such as *Ph. capsici* [27,64]. This observation is further supported by the role of RxLR and CRN effectors in other *Phytophthora* species, where large expansions of these effectors have been linked to pathogenic versatility and host adaptation [27,68]. *Phytophthora infestans* harbors over 500 predicted RxLR effectors [69], while other species like *Ph. capsici* contain 357 [64] and *Ph. sojae* 338 RxLR effectors [31]. The comparatively smaller number of effectors in *P. vexans* may suggest it employs a more specialized infection strategy, similar to species of *Pythium*, which rely on fewer but highly adapted RxLR and CRN effectors to target specific hosts [70].

*Phytophthora* species have evolved large repertoires of RxLRs and CRNs, and there is evidence that some of these effectors may be secreted through unconventional pathways not detected by traditional in silico methods [71]. This raises the possibility that *P. vexans* might also secrete additional, uncharacterized effectors through similar mechanisms, contributing to its pathogenicity despite the smaller number of identified effectors. Additionally, unique clusters of RxLR and CRN effectors in SS21 may reflect strain-specific adaptations that are essential for infection in its particular ecological niche or against specific host plants. Thus, although *P. vexans* contains fewer overall effectors, the diversity and uniqueness of the ones it retains might play a critical role in its pathogenic strategy, echoing findings in other oomycetes [27]. This reinforces the importance of effector diversity, genome architecture, and evolutionary adaptation in shaping the pathogenic potential of oomycetes, including *P. vexans*. However, the functional contributions of the predicted effectors identified in this study, whether cytoplasmic, apoplastic, or enzymatic, remain hypothetical. Further validation is needed to determine their specific roles in virulence, host specificity, or environmental adaptation.

## 5. Conclusions

Our in silico secretome analysis shows both variation and conservation in apoplastic and cytoplasmic effectors across *P. vexans* strains. Particularly, the HF1 strain harbors a higher abundance of key effector families, including CAZymes and necrosis-inducing proteins, which may contribute to enhanced plant cell wall degradation and broader pathogenic potential. In contrast, strain SS21, despite having fewer infection-related proteins, shows a unique set of effectors and CAZymes that may reflect specialized adaptations for pathogenicity in particular ecological or host-specific contexts. The conserved presence of protease inhibitors across all strains, along with the absence of phytotoxins and cutinases, suggests a selective and streamlined set of effectors that likely reflect distinct infection strategies.

This study provides, for the first time, an overview and comparison of the predicted secretome among three *P. vexans* strains. Although not exhaustive, it lays the groundwork for future research combining functional genomics and host–pathogen interaction studies to validate candidate effectors and further explain the molecular basis of *P. vexans* virulence and adaptation.

## Figures and Tables

**Table 1:** Assembly and annotation statistics of *P. vexans* genomes.

Genome Assembly	SS21	CBS 119.80	HF1
Sequencing technology	Illumina HiSeq	Illumina NextSeq	Illumina HiSeq and PacBio RS
Accession (NCBI)	JAKLCF000000000.2	JAADXV000000000.1	QLOC00000000.1
Genome size (Mbp)	35.3	33.85	41.73
Contigs number	2843	3685	44
Largest contig (bp)	124,174	185,880	3,173,098
Contig N50 (bp)	31,500	29,240	1,512,378
Contig N90 (bp)	5364	4392	634,848
GC content	59%	58.85%	58.17%
BUSCO genome completeness	99.0%	90.60%	92.20%
Genome coverage	412×	78×	87×
Predicted genes number	12,619	12,693	14,537
BUSCO protein completeness	99%	99%	99%

**Table 2:** The total number of predicted effector candidates identified across *P. vexans* strains SS21, CBS 119.80, and HF1. The numbers presented correspond to genes encoding proteins predicted to be secreted.

Category	Family	Number of Proteins Per Strain		
		SS21	CBS 119.80	HF1
MAMP	Sterol binding proteins	42	52	57
	Transglutaminase proteins	4	4	4
Apoplastic effectors	CAZymes **	89	107	146
	Glucanase inhibitors	6	6	12
	Phytotoxins	0	0	0
	Necrosis inducing proteins	28	28	52
	Cutinases	0	0	0
	Protease inhibitors	27	26	28
	Cysteine protease inhibitor	3	2	2
Cytoplasmic effectors	CRN *	85	100	112
	RXLR *	32	62	36

* Manually curated; ** CAZYme families obtained from dbCAN; MAMP: Microbe-Associated Molecular Pattern.

## Data Availability

The datasets generated and analyzed in this study are publicly available in the NCBI repository, [https://www.ncbi.nlm.nih.gov], under BioProject: PRJNA796576; BioSample ID: SAMN24899379; WGS project: JAKLCF02; GenBank assembly: GCA_022023675.2; Genome assembly: ASM2202367v2 and Sequence Read Archive (SRA): SRP451477.

## Supplementary Materials

The following supporting information can be downloaded at https://www.mdpi.com/article/10.3390/jof11070477/s1, Table S1. Core genes used to generate the phylogenetic tree and their corresponding functions and identifiers were sourced from the Saccharomyces Genome Database (SGD, www.yeastgenome.org) and UniProt (www.uniprot.org).
