# Supplementary material for: Profiling the Genomes and Secreted Effector Proteins in Phytopythium vexans Global Strains
Source: J Fungi (Basel). 2025 Jun 23;11(7):477. doi: 10.3390/jof11070477 (PMC12296172; doi:10.3390/jof11070477)
Supplement: Supplementary file 1 [file jof-11-00477-s001.zip › jof-3621923-supplementary.pdf]

Table S1. Core genes used to generate the phylogenetic tree and their corresponding functions and identifiers sourced from the *Saccharomyces* Genome Database (SGD, www.yeastgenome.org) and UniProt (www.uniprot.org).

Gene	Function	SGD	UniProt
ACT1	Actin	YFL039C	P60010
ATP6	F1F0 ATP synthase subunit	Q0085	P00854
BMS1	Ribosome biogenesis protein	YPL217C	Q08965
CCT8	Chaperonin-containing T-complex subunit	YJL008C	P47079
COB	Cytochrome b	Q0105	P00163
COX1	Cytochrome c oxidase subunit	Q0045	P00401
COX2	Cytochrome c oxidase subunit	Q0250	P00410
COX3	Cytochrome c oxidase subunit	Q0275	P00420
DIP2	U3 small nucleolar RNA-associated protein 12	YLR129W	Q12220
DPH5	Diphthine methyl ester synthase	YLR172C	P32469
DYS1	Deoxyhypusine synthase	YHR068W	P38791
ELP3	Elongator complex protein 3	YPL086C	Q02908
ESF1	Pre-rRNA-processing protein	YDR365C	Q06344
FAP7	Adenylate kinase isoenzyme 6 homolog	YDL166C	Q12055
FRS1	Phenylalanine--tRNA ligase beta subunit	YLR060W	P15624
HEM12	Uroporphyrinogen decarboxylase	YDR047W	P32347
HIS7	Imidazole glycerol phosphate synthase	YBR248C	P33734
ILV1	Threonine dehydratase	YER086W	P00927
KRE33	RNA cytidine acetyltransferase	YNL132W	P53914
MCM7	Mini-chromosome maintenance complex subunit	YBR202W	P38132
MET6	5-methyltetrahydropteroyltriglutamate--homocysteine methyltransferase	YER091C	P05694
MRPL19	54S ribosomal protein L19	YNL185C	P53875
MSF1	Phenylalanine--tRNA ligase	YPR047W	P08425
MVD1	Diphosphomevalonate decarboxylase	YNR043W	P32377
NOG1	Nucleolar GTP-binding protein 1	YPL093W	Q02892
PGK1	Phosphoglycerate kinase	YCR012W	P00560
POL2	DNA polymerase epsilon catalytic subunit A	YNL262W	P21951
PRT1	Eukaryotic translation initiation factor 3 subunit B	YOR361C	P06103
RAD2	DNA repair protein	YGR258C	P07276
RLI1	Translation initiation factor	YDR091C	Q03195
RPB2	DNA-directed RNA polymerase II core subunit	YOR151C	P08518
RPF2	Ribosome biogenesis protein	YKR081C	P36160

RPN1	26S proteasome regulatory subunit	YHR027C	P38764
RPO21	DNA-directed RNA polymerase II core subunit	YDL140C	P04050
RPP0	60S acidic ribosomal protein P0	YLR340W	P05317
SEC21	Coatomer subunit gamma	YNL287W	P32074
SEC26	Coatomer subunit beta	YDR238C	P41810
SPB1	27S pre-rRNA (guanosine(2922)-2'-O)-methyltransferase	YCL054W	P25582
TEF1	Translation elongation factor EF-1 alpha	YPR080W	P02994
TIF5	Eukaryotic translation initiation factor 5	YPR041W	P38431
TOP1	DNA topoisomerase 1	YOL006C	P04786
TRM1	tRNA (guanine(26)-N(2))-dimethyltransferase	YDR120C	P15565
TRP3	Multifunctional tryptophan biosynthesis protein	YKL211C	P00937
TSR1	Ribosome maturation factor	YDL060W	Q07381
TUB2	Beta-tubulin	YFL037W	P02557
UTP21	U3 small nucleolar RNA-associated protein 21	YLR409C	Q06078
VMA1	V-type proton ATPase catalytic subunit A	YDL185W	P17255
ZPR1	Zinc finger protein	YGR211W	P53303
